# Burden of undernutrition among under-five Bengali children and its determinants: Findings from Demographic and Health Surveys of Bangladesh and India

**DOI:** 10.1371/journal.pone.0301808

**Published:** 2024-04-05

**Authors:** Ramendra Nath Kundu, Md. Golam Hossain, Md. Ahshanul Haque, Rashidul Alam Mahumud, Manoranjan Pal, Premananda Bharati

**Affiliations:** 1 Department of Anthropology, Former Senior Research Fellow (UGC-NET), West Bengal State University, West Bengal, India; 2 Department of Statistics, Health Research Group, University of Rajshahi, Rajshahi, Bangladesh; 3 Nutrition and Clinical Services Division, icddr,b, Dhaka, Bangladesh; 4 Faculty of Medicine and Health, NHMRC Clinical Trials Centre, The University of Sydney, Camperdown, New South Wales, Australia; 5 Indian Statistical Institute, Economic Research Unit, Kolkata, West Bengal, India; 6 Indian Statistical Institute, Biological Anthropology Unit, Kolkata, West Bengal, India; University of Michigan, UNITED STATES

## Abstract

**Background:**

Globally, undernutrition is the leading cause of mortality among under-five children. Bangladesh and India were in the top ten countries in the world for under-five mortality. The aim of the study was to investigate the nutritional status of Bengali under-five children.

**Methods:**

Data on 25938 under-five children were retrieved from the Bangladesh Demographic and Health Survey 2017–18 (BDHS) and the National Family Health Survey of India 2015–16 (NFHS-4). Stunting, wasting, underweight and thinness were considered to understand the nutritional status of under-five children. Binary logistic regression was used to identify associated factors of undernutrition among children.

**Results:**

Over one-quarter of Bengali under-five children were found to be suffering from the problem of stunting (31.9%) and underweight (28.1%), while other nutritional indicators raised serious concern and revealed inter-country disparities. In the cases of wasting, underweight and thinness, the mean z-scores and frequency differences between Bangladesh and India were significant. The nutritional status of Bengali under-five children appeared to have improved in Bangladesh compared to India. Child undernutrition had significant relations with maternal undernutrition in both countries. Girls in Bangladesh had slightly better nutritional status than boys. In Bangladesh, lack of formal education among mothers was a leading cause of child undernutrition. Stunting and underweight coexist with low household wealth index in both counties.

**Conclusions:**

The research revealed that various factors were associated with child undernutrition in Bengalis. It has been proposed that programmes promoting maternal education and nutrition, along with household wealth index be prioritised. The study recommends that the Governments of Bangladesh and India should increase the budget for health of children so as to reach the sustainable development goals.

## Introduction

Undernutrition is the leading cause of mortality for nearly half of all under-five child deaths worldwide. It increases the chance of morbidity, puts children at risk of dying from common diseases, increases the severity of infections, and delays recovery [1]. At the same time, the United Nations Sustainable Development Goals (SDGs) have set a target of eradicating hunger by 2030 [2,3]. In 2020, globally 149.2 million children under the age of five were stunted and 45.4 million wasted, with Asian countries accounting for 79 million being stunted and 31.9 million being wasted [4]. Over the past two decades, undernutrition has emerged as a major child health concern in low-and-middle-income countries (LMICs) due to its strong association with child mortality [5]. The same tendency can be seen in Bangladesh and India, being the large and high density two LMICs in South Asia. According to the World Health Organization (WHO), India ranked second (824000 deaths) and Bangladesh ranked tenth (90000 deaths) among the top ten countries considering the highest number of under-five mortality in 2019 [6].

In Bangladesh, Bengalis constitute the majority of population, whereas, in India, the Bengalis are the second-largest linguistic community (8.03%), with majority of them living in Tripura and West Bengal [7]. According to the 2011 census, over 241 million Bengalis were living in these two countries, with 144.04 million in Bangladesh and 97.24 million in India [7,8]. Historically this population shared a common culture, despite their religious variation, as they are descendants of the Indo-Aryan branch of the Indo-European linguistic family. They also have a similar environment in terms of ecology, food patterns, and socioeconomic background in these two countries.

Nutritional status is a multifactorial trait; non-genetic factors such as dietary habits, socioeconomic condition, and environmental factors influence nutritional status along with inheritance [9]. Many studies have found that malnutrition can be reduced by controlling those non-genetic factors so that the heredity cannot be controlled in general [10,11]. Several studies on the nutritional status of children in Bangladesh and India have been carried out independently in both countries [12–17]. Most of these studies have limitations because they focus on a specific region, not to the whole population. In order to understand it in a better way, a comparative study is required on the issues that affect the entire Bengali population across the country in the present context. This will help both the countries undertake nutritional development programmes to achieve the UN’s Sustainable Development Goals–no poverty, zero hunger, good health and well-being, and reduced inequality [2].

The focus of this study is to determine the prevalence of under-five child undernutrition, such as stunting, wasting, underweight, and thinness, among Bengali children in Bangladesh and India. Also, it is aimed to investigate the impact of child-related, maternal and socio-demographic factors on the health conditions of the children. Such data would help us to prevent and regulate child malnutrition in the Bengali community of both the countries.

## Methods

### Data source

**Fig 1:**
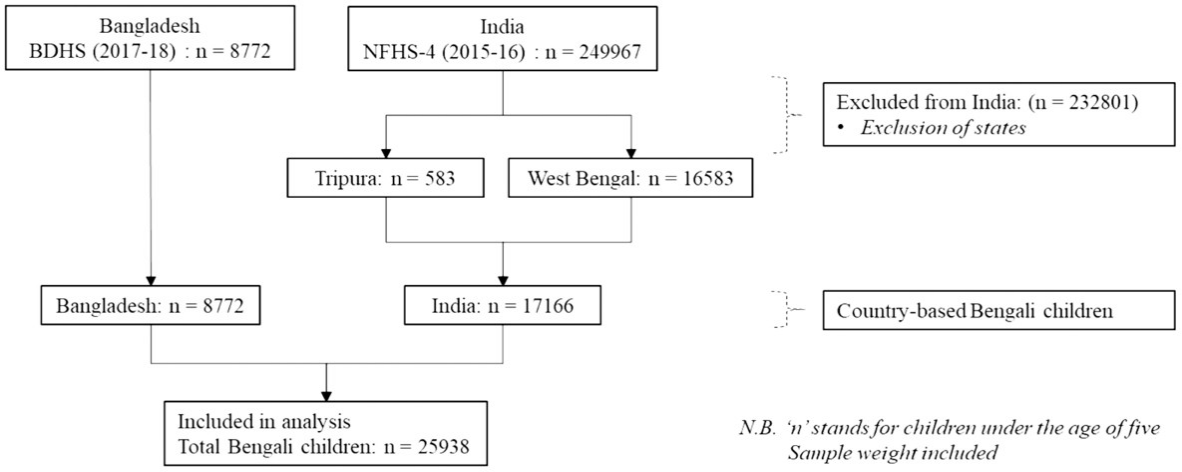
Sample selection of under-five Bengali children for the statistical analysis.

The unit-level data for this study were taken from the Bangladesh Demographic and Health Survey 2017–18 (BDHS) and the National Family Health Survey of 2015–16 (NFHS-4), which were publicly available. The BDHS and NFHS were national household surveys in Bangladesh and India, respectively, which used standardized questionnaires, sampling designs, and field methodologies to comply with Demographic and Health Surveys (DHS) principles. A recognized nodal organization, such as the National Institute of Population Research and Training (NIPORT) for the BDHS and the International Institute for Population Sciences (IIPS) for the NFHS, evaluated all survey protocols for each country. Based on the official language, Tripura and West Bengal were considered the states that best represented the Bengali people in India. A total of 25938 under-five children were included for statistical analysis, with the flow chart in Fig 1 illustrating data inclusion and exclusion.

### Unit level study variables

#### Outcome variable

The outcome variables were stunting, wasting, underweight, and thinness of the under-five children. According to the WHO (2006), a z-score less than -2SD of height-for-age was considered as stunting, weight-for-height was considered as wasting, weight-for-age was considered as underweight, and BMI-for-age was considered as thinness [18]. Children with adequate height, weight, and BMI (z-score ≥ -2SD) were considered as non-undernourished. The data was analyzed using a dichotomous category for child nutrition, with a z-score of < -2SD for target dependent groups and ≥ -2SD for reference dependent groups.

#### Explanatory variables

Child-related, maternal, and socio-demographic factors were included in the study as the possible determinants of stunting, wasting, underweight, and thinness. Explanatory variables were classified using previously published articles [19–24]. The child-related factors includes, child’s gender (boy, girl), age of children (0–23 months, 24–59 months), and birth order number (first, second, third & more). The child’s age was classified based on breastfeeding duration, with a range of 0–23 months for breastfeeding period and 24–59 months for post-breastfeeding period. The maternal factors includes, Body Mass Index (BMI), which was classified based on the WHO (2004) suggested cut-off of <18.5 for underweight, 18.5 to 24.9 for normal, 25 to 29.9 for overweight, and ≥30 for obese [25]. Age of mother (15–17, 18–34, 35–49 years), education of mother (no education, primary, secondary, higher), mode of delivery (vaginal, caesarean), and antenatal care visits (no antenatal visit, less than four times, four times & above). The variables of socio-demographic factors includes, residence (urban, rural), religion (Hindu, Muslim, Christian & Buddhist), household size (up to three, four, five, more than five), and wealth index (poorest, poorer, middle, richer, richest). Due to low frequency (<5% of population), Christians and Buddhists were merged in a single group.

### Ethical approval

This study used secondary data from the Demographic and Health Surveys (DHS). The ICF Institutional Review Board (IRB) and the host country’s DHS ethical committee (NIPORT in Bangladesh and IIPS in India) have both reviewed and approved the survey procedures and participants confidentiality. The ICF IRB adheres to the US Department of Health and Human Services’ guidelines for the protection of human subjects and participants confidentiality. Also, DHS received written consent from legal guardians of each selected subjects. The DHS data was ethically approved, and its use did not require any further ethical approval.

### Statistical analysis

Mean and standard deviation (SD) for quantitative variables, also frequencies and percentages for qualitative variables were calculated as summary statistics. The testing for differences of means between the countries was done using statistics which followed t- distribution, while Z-test was used for testing the differences in proportions of frequency. It should be noted here that for large sample size t-test was almost same as Z-test. Graphical comparisons of nutritional status between the studied population and WHO-standard were processed by WHO-Anthro Survey Analyser. Binary logistic regression was used to find significant factors of undernutrition indicators such as stunting, wasting, underweight, and thinness as dependent variable. Undernourished group was given a code ‘1’, against non-undernourished as ‘0’. Independent variables were chosen after a multicollinearity test, and the variance inflation factor (VIF) was shown to be less than 5. The p-value was considered significant at 0.05. The Statistical Package for the Social Sciences was used to analyze the data (SPSS, version 25.0).

## Results

A total of 25938 data were included (Bangladesh 8772 and India 17166), with 51.7% of boys and 48.3% of girls distributed almost evenly. The mean age of the children was 28.7 (SD 17.5) months in Bangladesh and 30.0 (SD 17.1) months in India. Table 1A shows the distribution of mean z-scores for height-for-age (HAZ), weight-for-height (WHZ), weight-for-age (WAZ), and BMI-for-age (BAZ). In terms of WHZ, WAZ, and BAZ, there were substantial differences between Bangladesh and India, but not in HAZ. Table 1B, on the other hand, indicates the prevalence of all kinds of undernutrition reported in Indian children. Although both countries faced major issues with childhood stunting and underweight, the prevalence of underweight was higher in India (31.3%) than in Bangladesh (21.8%). Except for stunting, the frequency distribution of each undernourished group differed significantly between countries, with p values <0.001 (Table 1A and 1B).

**Table 1:** **A:** Results of differences in mean z-scores of nutritional indicators of Bengali children between Bangladesh and India. **B:** Results of differences in the proportions of undernutrition between under-five Bengali children in India and Bangladesh.

Nutritional indicator	Overall Mean (SD)	Bangladesh Mean (SD)	India Mean (SD)	t-value (BD-IN)	p-value	Nutritional indicator	Overall No. (%)	Bangladesh No. (%)	India No. (%)	Z-value (BD-IN)	p-value
HAZ	-1.36 (1.39)	-1.37 (1.32)	-1.36 (1.43)	-0.884	0.376	Stunting	7439 (31.86)	2402 (30.72)	5038 (32.43)	-1.48	0.139
WHZ	-0.83 (1.29)	-0.53 (1.16)	-0.98 (1.33)	25.42	<0.001	Wasting	3776 (16.18)	659 (8.44)	3117 (20.07)	-7.05	<0.001
WAZ	-1.35 (1.16)	-1.16 (1.12)	-1.45 (1.17)	18.49	<0.001	Underweight	6616 (28.07)	1753 (21.80)	4863 (31.31)	-7.54	<0.001
BAZ	-0.72 (1.31)	-0.41 (1.16)	-0.87 (1.35)	25.44	<0.001	Thinness	3311 (14.19)	559 (7.16)	2751 (17.71)	-6.21	<0.001

**Fig 2:**
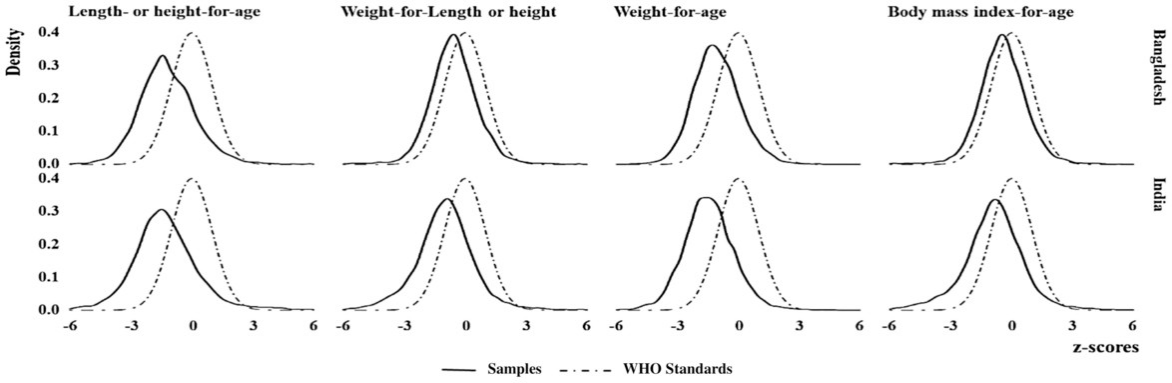
Comparison of nutritional status among children across countries using z-score distribution of the WHO standards.

The nutritional status of Bengali children (simple line) was compared with that of the WHO-standards (dotted line) in Fig 2. Although, all the nutritional indicators showed that nutritional status was normally distributed, their mean z-score values were much lower than the WHO-standard z-score values. All of the analyzed curves of Bengali children were on the negative side of the WHO-standard reference curve, indicating poor nutritional status in both Bangladesh and India. HAZ and WAZ were approximately evenly distributed across both countries; however WHZ and BAZ in Bangladesh were much closer to WHO than in India. HAZ was mesokurtic and the density in Bangladesh was little more than India, with distributed negative to the WHO-standard reference curve. In comparison to India, the curve for WHZ and BAZ in Bangladesh was normal, and their mean z-score value was closer to the WHO standard z-score values (Fig 2).

**Fig 3:**
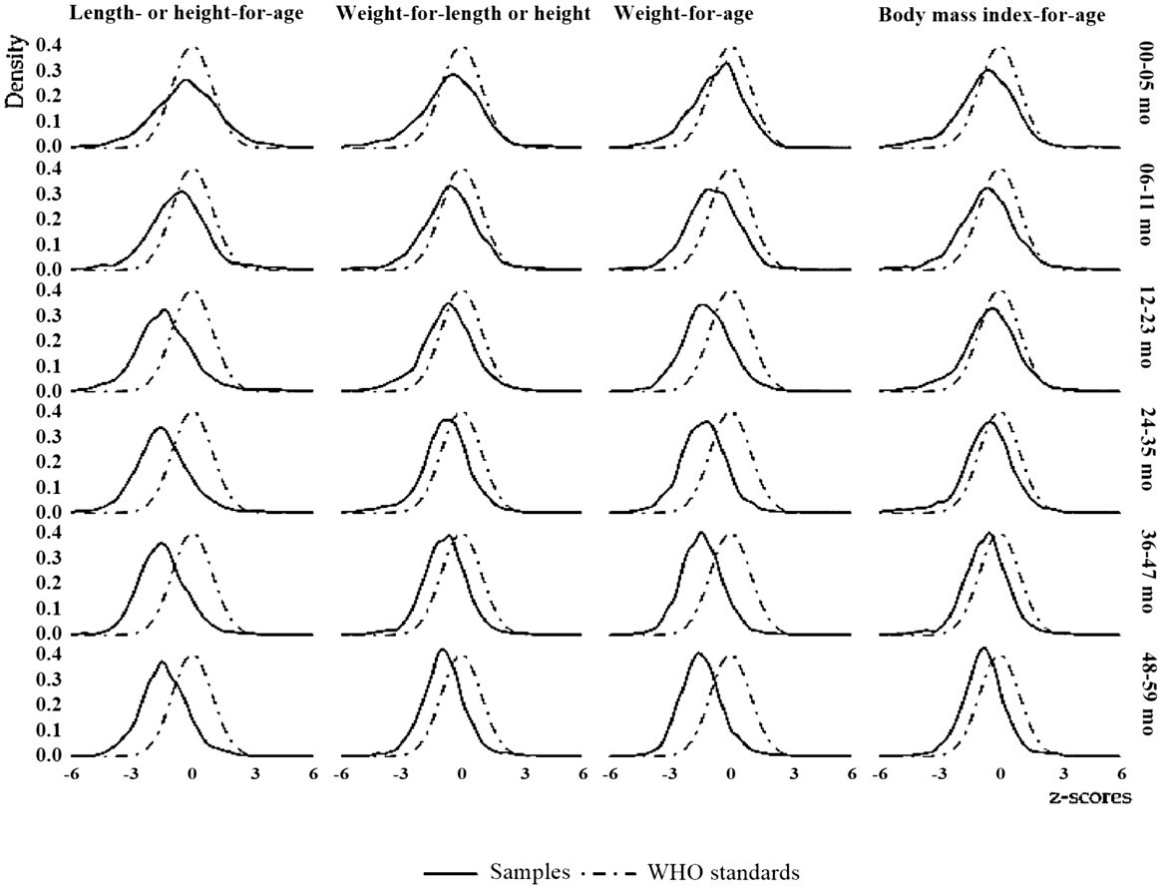
Distribution of z-score by indicators and age group among overall Bengali children.

Changes in the age-based nutritional status of overall Bengali children were shown in Fig 3. The plots were almost asymmetrical and the tails were quite dense. A similarity was found between HAZ and WAZ, both of which had plots in the first 5 months observed in WHO-standards. WHZ and BAZ were found to be comparable, in terms of age groups, both plots were marginally closer to the WHO-standard, although the tail density was more prominent in the early age groups, which had mesokurtic distributions and virtually asymmetric distributions. The tail density reduces with increasing age in all plots, showing ethnic proximity. According to HAZ and WAZ, children became undernourished as they grew older, and their height and weight were far below WHO-standards. BAZ denotes that BMI was lower than age, WHZ indicates that body mass was lower than height as per WHO-standards (Fig 3).

### Explanatory factors and its distribution

Table 2 shows that the frequency of the types of explanatory factors differed significantly (Z-value, p <0.05) between Bangladesh and India, with the exception of the gender, age, residence and household size. Although, the percentage gap between boys and girls was higher in Bangladesh (4.48) than in India (2.78), while boys outnumbered girls in both countries. Single children were more common in Bengalis, accounting for 45.88% of the population, while the proportion was somewhat higher in India (49.55%). Maternal undernutrition was more prevalent in both nations than maternal over nutrition, with India having the most undernourished mothers. In Bangladesh, 12.97% of mothers gave birth to a child before 18, and the percentage was higher than in India. One out of every seven Bengali mothers was uneducated, compared to one out of every sixth in India. Caesarean births were more common, accounting for one out of every four births in Bangladesh than one out of every three in India. In Bangladesh, more than half of the mothers do not visit more than three times to the health centre for their antenatal care (ANC). In Bengali, more than half of the children were Muslims, while 91.96% in Bangladesh. About 20.81% of households in both countries had up to five members in the household. Among the Bengalis, more than half of the households have a low wealth index, while the proportion was somewhat higher in India (Table 2).

**Table 2:** Classification of explanatory factors and its distribution by country among under-five Bengali children.

Explanatory Factors	Overall	Bangladesh	India	Z-value*
	No. (%)	No. (%)	No. (%)	(p-value)
Child’s gender				
Boy	13404 (51.68)	4583 (52.24)	8821 (51.39)	0.93 (0.352)
Girl	12534 (48.32)	4189 (47.76)	8345 (48.61)	-0.90 (0.368)
Age of children				
0–23 months	10067 (40.52)	3456 (41.53)	6611 (40.01)	1.47 (0.142)
24–59 months	14779 (59.48)	4865 (58.47)	9914 (59.99)	-1.77 (0.077)
Birth order number				
First	11901 (45.88)	3395 (38.70)	8506 (49.55)	-10.72 (<0.001)
Second	8306 (32.02)	2800 (31.92)	5506 (32.08)	-0.15 (0.881)
Third & more	5731 (22.10)	2577 (29.38)	3154 (18.38)	9.80 (<0.001)
Mother’s BMI				
Underweight	5373 (21.10)	1196 (13.87)	4177 (24.80)	-8.00 (<0.001)
Normal	15493 (60.84)	5202 (60.33)	10291 (61.10)	-0.93 (0.352)
Overweight	3699 (14.53)	1777 (20.62)	1922 (11.41)	7.67 (<0.001)
Obese	899 (3.53)	447 (5.18)	452 (2.68)	1.93 (0.053)
Age of mother				
15–17 years	2478 (9.55)	1138 (12.97)	1340 (7.80)	4.24 (<0.001)
18–34 years	21858 (84.27)	6949 (79.22)	14909 (86.85)	14.49 (<0.001)
35–49 years	1602 (6.18)	685 (7.81)	917 (5.34)	2.00 (<0.045)
Education of mother				
No education	3622 (13.97)	645 (7.36)	2977 (17.34)	-6.34 (<0.001)
Primary	6228 (24.01)	2528 (28.82)	3700 (21.56)	6.54 (<0.001)
Secondary	13674 (52.72)	4248 (48.43)	9426 (54.91)	-7.02 (<0.001)
Higher	2413 (9.30)	1351 (15.40)	1062 (6.19)	7.08 (<0.001)
Mode of delivery				
Vaginal	16682 (74.15)	3584 (67.23)	13098 (76.30)	-11.02 (<0.001)
Caesarean	5815 (25.85)	1747 (32.77)	4068 (23.70)	7.19 (<0.001)
Antenatal care visits				
No antenatal visits	1650 (8.47)	405 (8.01)	1245 (8.63)	-0.39 (0.697)
Less than Four times	4358 (22.37)	2272 (44.98)	2086 (14.45)	21.89 (<0.001)
Four times and above	13475 (69.16)	2374 (47.01)	11101 (76.92)	29.35 (<0.001)
Residence				
Urban	7173 (27.65)	2413 (27.51)	4760 (27.73)	0.20 (0.841)
Rural	18765 (72.35)	6359 (72.49)	12406 (72.27)	0.32 (0.749)
Religion				
Hindu	11632 (45.94)	648 (7.39)	10984 (66.39)	-30.25 (<0.001)
Muslim	13507 (53.35)	8067 (91.96)	5440 (32.88)	72.29 (<0.001)
Christian & Buddhist	179 (0.71)	57 (0.65)	122 (0.73)	-0.06 (<0.952)
Household size				
Upto three	3241 (12.49)	1169 (13.33)	2072 (12.07)	1.04 (0.298)
Four	5497 (21.19)	1779 (20.28)	3718 (21.66)	-1.17 (0.242)
Five	5397 (20.81)	1736 (19.79)	3661 (21.33)	-1.30 (0.194)
More than five	11805 (45.51)	4089 (46.61)	7716 (44.95)	1.72 (0.085)
Wealth index				
Poorest	6366 (24.54)	1884 (21.47)	4482 (26.11)	-3.92 (<0.001)
Poorer	7544 (29.09)	1780 (20.29)	5764 (33.58)	-10.65 (<0.001)
Middle	5111 (19.70)	1647 (18.77)	3464 (20.18)	-1.18 (0.238)
Richer	4171 (16.08)	1764 (20.11)	2407 (14.02)	5.22 (<0.001)
Richest	2745 (10.59)	1697 (19.35)	1048 (6.11)	9.63 (<0.001)

*proportional Z-test between Bangladesh and India.

### Predictors of under nutrition in overall Bengali children

We observed that the VIF value of each independent variable was less than 5, there was no evidence of multicollinearity problem among independent variables for stunting, wasting, underweight and thinness. After controlling the effect of other factors, multiple logistic regression model demonstrated that girls were less likely to get wasting (p<0.01) and thinness (p<0.05) compared to boys for overall Bengali children. Older (age, 24–59 months) children had a 1.38 and 1.62-folds higher chance to get stunting (p<0.01) and underweight (p<0.01) respectively compared to younger (age, 0–23 months) children, while older children was less likely to have thinness (p<0.01) than younger children. Children who were born in later had a 1.21 (p<0.01) (birth order second) and 1.17-folds (p<0.01) (birth order third & more) higher chance to have stunting compared to birth order first, while third or more order children were less likely to get wasting than first order (p<0.05). Underweight mother’s son/daughter was 1.20 (p<0.01), 1.73 (p<0.01), 1.91 (p<0.01) and 1.76 (p<0.01) times more chance to have stunting, wasting, underweight and thinness respectively compared to normal weight mother’s son/daughter, while opposite results were found for overweight and obese mothers. Younger (age, 15–17 years) mothers’ children were less likely to get wasting (p<0.05) and underweight (p<0.05) than older (age, 35–49 years) mothers’ children. Less than higher educated mothers’ children were more likely to have stunting, wasting, underweight and thinness than compared to higher educated mothers’ children (p<0.01). Caesarean delivered children were less likely to get stunting, wasting, underweight and thinness than vaginal delivered children (p<0.01). Mothers received less than four times antenatal care (ANC) who had less chance to get wasting (p<0.01) and thinness (p<0.01) children compared mothers received four or more times ANC. Muslim children was more likely to have stunting (p<0.01) but less chance to have wasting (p<0.01), underweight (p<0.05) and thinness (p<0.01) compared to Hindu children. We found that children living in a household consisting four members who had less chance to get stunting (p<0.01) and underweight (p<0.01) compared to children living in a household having upto three members. Children living in poorest, poorer, middle and rich family were more likely to have stunting and underweight than children living in richest family (p<0.01), also children in poorest family was more chance to get wasting than children in richest family (p<0.01) (Table 3).

**Table 3:** The result of binary logistic regression of child’s nutrition indicators on the explanatory factors among the overall Bengalis.

Explanatory Factors	Stunting	Wasting	Underweight	Thinness
	AOR (95% CI)	AOR (95% CI)	AOR (95% CI)	AOR (95% CI)
Child’s gender (Ref. Boys)				
Girls	0.99 (0.93, 1.06)	0.88 (0.81, 0.96)**	1.00 (0.94, 1.08)	0.91 (0.84, 0.99)*
Age of children (Ref. 0–23 months)				
24–59 months	1.38 (1.29, 1.48)**	0.96 (0.88, 1.04)	1.62 (1.50, 1.74)**	0.80 (0.73, 0.87)**
Birth order number (Ref. First)				
Second	1.21 (1.11, 1.32)**	0.95 (0.86, 1.06)	1.09 (1.00, 1.20)	0.91 (0.82, 1.02)
Third & more	1.17 (1.05, 1.30)**	0.84 (0.74, 0.96)*	1.06 (0.95, 1.19)	0.92 (0.80, 1.05)
Mother’s BMI (Ref. Normal)				
Underweight	1.20 (1.10, 1.30)**	1.73 (1.57, 1.89)**	1.91 (1.76, 2.07)**	1.76 (1.60, 1.94)**
Overweight	0.95 (0.85, 1.05)	0.71 (0.62, 0.82)**	0.70 (0.62, 0.79)**	0.74 (0.64, 0.86)**
Obese	0.53 (0.42, 0.68)**	0.44 (0.31, 0.63)**	0.45 (0.34, 0.60)**	0.58 (0.41, 0.80)**
Age of mother (Ref. 35–49 years)				
15–17 years	1.03 (0.85, 1.24)	0.77 (0.61, 0.98)*	0.79 (0.64, 0.96)*	0.86 (0.67, 1.11)
18–34 years	1.05 (0.91, 1.22)	0.97 (0.80, 1.17)	0.87 (0.75, 1.02)	1.05 (0.86, 1.29)
Education of mother (Ref. Higher)				
No education	1.85 (1.55, 2.20)**	1.58 (1.26, 1.97)**	2.26 (1.85, 2.76)**	1.65 (1.31, 2.08)**
Primary	1.71 (1.45, 2.01)**	1.42 (1.15, 1.76)**	1.82 (1.51, 2.20)**	1.37 (1.10, 1.71)**
Secondary	1.28 (1.10, 1.49)**	1.42 (1.18, 1.72)**	1.57 (1.32, 1.86)**	1.45 (1.19, 1.77)**
Mode of delivery (Ref. Vaginal)				
Caesarean	0.87 (0.80, 0.94)**	0.65 (0.58, 0.73)**	0.71 (0.65, 0.78)**	0.70 (0.63, 0.79)**
No antenatal visits	1.08 (0.96, 1.22)	1.01 (0.87, 1.16)	1.04 (0.92, 1.17)	1.14 (0.98, 1.32)
Less than four times	1.01 (0.93, 1.10)	0.76 (0.69, 0.85)**	0.95 (0.87, 1.04)	0.80 (0.72, 0.90)**
Residence (Ref. Urban)				
Rural	0.97 (0.89, 1.06)	0.98 (0.89, 1.09)	0.92 (0.84, 1.00)	1.04 (0.93, 1.16)
Religion (Ref. Hindu)				
Muslim	1.34 (1.25, 1.44)**	0.66 (0.60, 0.72)**	0.91 (0.84, 0.98)*	0.59 (0.53, 0.64)**
Christian & Buddhist	0.86 (0.56, 1.33)	0.83 (0.51, 1.37)	0.76 (0.49, 1.20)	0.82 (0.49, 1.38)
Household size (Ref. Upto three)				
Four	0.81 (0.71, 0.92)**	0.91 (0.78, 1.06)	0.81 (0.71, 0.93)**	0.96 (0.82, 1.13)
Five	1.04 (0.92, 1.17)	0.96 (0.83, 1.12)	0.93 (0.82, 1.06)	0.98 (0.84, 1.15)
More than five	0.98 (0.88, 1.10)	0.96 (0.84, 1.10)	0.97 (0.86, 1.08)	1.04 (0.90, 1.19)
Wealth index (Ref. Richest)				
Poorest	2.03 (1.71, 2.41)**	1.43 (1.15, 1.77)**	2.20 (1.82, 2.66)**	1.13 (0.90, 1.40)
Poorer	1.73 (1.47, 2.04)**	1.19 (0.96, 1.46)	1.67 (1.39, 2.01)**	1.01 (0.82, 1.25)
Middle	1.74 (1.48, 2.05)**	0.95 (0.77, 1.16)	1.29 (1.07, 1.55)**	0.82 (0.66, 1.01)
Richer	1.24 (1.06, 1.46)**	1.17 (0.96, 1.43)	1.23 (1.03, 1.48)**	1.02 (0.84, 1.26)

**N.B.**:

**p<0.01,

*p<0.05.

### Predictors of child undernutrition in Bangladesh and India separately

Wasting was less likely to be among girls in both countries, while thinness was more prevalent among girls in Bangladesh compared to boys. Stunting and underweight were more likely to be found in children after the end of the breastfeeding phase in both countries, although wasting and thinness were more prevalent in India throughout the breastfeeding period. Birth order have relatively less effect on child undernutrition, in India, the second child was more likely to suffer from stunting and underweight whereas in Bangladesh, the second child was less likely to suffer from stunting than the first child. Undernutrition among children was more prevalent among underweight mothers, and child and maternal undernutrition coexisted in both nations. In India, younger mothers were less likely to have underweight children than older mothers. In Bangladesh, children with lower maternal education were twice as likely to be undernourished, whereas stunting and underweight children were more prevalent in India. Children had caesarean deliveries were less likely to be undernourished; stunting, and underweight in Bangladesh, whereas wasting, underweight, and thinness in India. In Bangladesh, stunting and underweight were more likely to have the mothers who did not get any ANC, while in India only thinness was more prevalent. Children in rural areas were 1.14 times more likely to have thinness than in urban areas in India. Muslim children in India were more likely to suffer from stunting and less likely to suffer from wasting and thinness than Hindu children; while Christian & Buddhist children in Bangladesh were more likely to suffer from wasting and thinness. Children of households with four members were 26 percent less likely in India and 30 percent less likely in Bangladesh so far as suffering from stunting and underweight respectively were concerned compared to three-member households. Stunting and underweight were more prevalent in children from poor wealth index families in both countries, although wasting in India and thinness in Bangladesh were less prevalent in poor wealth index families (Table 4).

**Table 4:** The result of binary logistic regression of child’s nutrition indicators on the explanatory factors among under-five Bengali children separately for Bangladesh and India.

Explanatory Factors	Stunting		Wasting		Underweight		Thinness	
	Bangladesh	India	Bangladesh	India	Bangladesh	India	Bangladesh	India
	AOR (95% CI)	AOR (95% CI)	AOR (95% CI)	AOR (95% CI)	AOR (95% CI)	AOR (95% CI)	AOR (95% CI)	AOR (95% CI)
Child’s gender (Ref. Boys)								
Girls	0.93 (0.82, 1.06)	1.01 (0.94, 1.10)	0.77 (0.62, 0.96)*	0.90 (0.82, 0.98)*	0.88 (0.75, 1.02)	1.04 (0.96, 1.13)	0.75 (0.60, 0.94)*	0.94 (0.86, 1.03)
Age of children (Ref. 0–23 months)								
24–59 months	1.83 (1.59, 2.11)***	1.25 (1.16, 1.36)***	1.04 (0.82, 1.31)	0.87 (0.80, 0.96)**	1.75 (1.50, 2.05)***	1.52 (1.40, 1.66)***	0.78 (0.60, 1.01)	0.74 (0.67, 0.81)***
Birth order number (Ref. First)								
Second	0.81 (0.68, 0.98)*	1.36 (1.23, 1.51)***	1.02 (0.76, 1.37)	0.97 (0.86, 1.08)	0.96 (0.77, 1.18)	1.12 (1.01, 1.24)*	1.10 (0.80, 1.50)	0.92 (0.81, 1.03)
Third & more	0.88 (0.71, 1.08)	1.26 (1.11, 1.43)***	0.71 (0.50, 1.01)	0.94 (0.81, 1.09)	0.98 (0.77, 1.24)	1.10 (0.97, 1.25)	0.92 (0.64, 1.33)	1.00 (0.86, 1.16)
Mother’s BMI (Ref. Normal)								
Underweight	1.28 (1.07, 1.53)**	1.16 (1.06, 1.27)**	1.95 (1.50, 2.53)***	1.64 (1.49, 1.82)***	1.68 (1.39, 2.04)***	1.91 (1.74, 2.09)***	1.82 (1.38, 2.41)***	1.70 (1.54, 1.89)***
Overweight	0.94 (0.77, 1.13)	0.96 (0.84, 1.09)	1.11 (0.81, 1.50)	0.65 (0.55, 0.77)***	0.77 (0.61, 0.97)*	0.68 (0.59, 0.78)***	1.06 (0.77, 1.46)	0.69 (0.58, 0.82)***
Obese	0.88 (0.61, 1.27)	0.40 (0.29, 0.56)***	0.55 (0.27, 1.15)	0.41 (0.27, 0.61)***	0.68 (0.43, 1.08)	0.37 (0.26, 0.53)***	0.68 (0.34, 1.34)	0.55 (0.37, 0.80)**
Age of mother (Ref. 35–49 years)								
15–17 years	0.96 (0.67, 1.38)	0.94 (0.74, 1.18)	0.89 (0.49, 1.63)	0.85 (0.65, 1.11)	1.03 (0.68, 1.55)	0.72 (0.57, 0.91)**	1.54 (0.79, 2.98)	0.86 (0.65, 1.14)
18–34 years	1.06 (0.78, 1.44)	1.03 (0.87, 1.23)	0.93 (0.56, 1.54)	1.03 (0.84, 1.26)	1.02 (0.73, 1.43)	0.84 (0.71, 1.00)	1.26 (0.71, 2.23)	1.07 (0.86, 1.32)
Education of mother (Ref. Higher)								
No education	2.01 (1.43, 2.83)***	1.59 (1.27, 2.00)***	2.50 (1.49, 4.18)***	1.16 (0.89, 1.52)	2.54 (1.73, 3.73)***	1.90 (1.47, 2.45)***	2.36 (1.39, 4.00)**	1.27 (0.96, 1.68)
Primary	2.10 (1.62, 2.71)***	1.44 (1.16, 1.80)**	1.56 (1.05, 2.34)*	1.23 (0.95, 1.59)	1.92 (1.42, 2.59)***	1.68 (1.31, 2.15)***	1.25 (0.82, 1.89)	1.25 (0.95, 1.63)
Secondary	1.74 (1.38, 2.19)***	1.06 (0.87, 1.30)	1.36 (0.95, 1.93)	1.20 (0.95, 1.52)	1.64 (1.25, 2.15)***	1.43 (1.13, 1.80)**	1.28 (0.89, 1.83)	1.27 (1.00, 1.63)
Mode of delivery (Ref. Vaginal)								
Caesarean	0.74 (0.63, 0.87)***	0.93 (0.84, 1.03)	0.80 (0.62, 1.04)	0.64 (0.57, 0.73)***	0.79 (0.65, 0.95)*	0.70 (0.63, 0.78)***	0.87 (0.67, 1.14)	0.69 (0.60, 0.78)***
Antenatal care visits (Ref. Four times and above)								
No antenatal visits	1.31 (1.02, 1.68)*	1.02 (0.89, 1.17)	0.73 (0.47, 1.14)	1.11 (0.95, 1.29)	1.32 (1.01, 1.73)*	1.01 (0.87, 1.16)	0.80 (0.51, 1.25)	1.26 (1.08, 1.48)**
Less than Four times	1.10 (0.95, 1.27)	0.95 (0.85, 1.06)	0.93 (0.74, 1.18)	0.89 (0.78, 1.01)	1.01 (0.85, 1.19)	1.04 (0.93, 1.17)	0.88 (0.68, 1.12)	0.96 (0.84, 1.10)
Residence (Ref. Urban)								
Rural	1.02 (0.86, 1.21)	0.96 (0.86, 1.06)	0.81 (0.62, 1.06)	1.09 (0.97, 1.22)	0.90 (0.74, 1.10)	0.93 (0.84, 1.03)	0.89 (0.68, 1.18)	1.14 (1.01, 1.29)*
Religion (Ref. Hindu)								
Muslim	1.18 (0.92, 1.52)	1.33 (1.22, 1.45)***	1.51 (0.95, 2.42)	0.81 (0.73, 0.89)***	1.04 (0.78, 1.39)	0.98 (0.90, 1.07)	1.58 (0.95, 2.62)	0.70 (0.63, 0.78)***
Christian & Buddhist	0.75 (0.29, 1.96)	0.88 (0.53, 1.44)	4.15 (1.39, 12.4)*	0.73 (0.41, 1.30)	0.89 (0.33, 2.44)	0.79 (0.48, 1.31)	4.6 (1.46, 14.45)**	0.71 (0.39, 1.30)
Household size (Ref. Upto three)								
Four	1.01 (0.79, 1.30)	0.74 (0.63, 0.85)***	0.70 (0.47, 1.04)	0.97 (0.82, 1.14)	0.70 (0.53, 0.93)*	0.86 (0.74, 1.00)	0.92 (0.59, 1.44)	0.99 (0.83, 1.18)
Five	1.06 (0.83, 1.36)	1.02 (0.89, 1.18)	1.08 (0.73, 1.58)	0.95 (0.80, 1.12)	0.79 (0.60, 1.04)	0.98 (0.84, 1.13)	1.30 (0.85, 1.99)	0.94 (0.80, 1.12)
More than five	0.96 (0.77, 1.19)	0.98 (0.86, 1.12)	0.93 (0.67, 1.30)	0.98 (0.85, 1.14)	0.83 (0.65, 1.05)	1.03 (0.90, 1.17)	1.20 (0.82, 1.76)	1.02 (0.88, 1.19)
Wealth index (Ref. Richest)								
Poorest	1.72 (1.31, 2.24)***	2.32 (1.82, 2.97)***	1.01 (0.67, 1.52)	1.51 (1.14, 1.99)**	1.46 (1.07, 1.98)*	2.75 (2.11, 3.58)***	0.95 (0.62, 1.44)	1.16 (0.87, 1.53)
Poorer	1.70 (1.31, 2.21)***	1.93 (1.53, 2.45)***	0.81 (0.54, 1.22)	1.20 (0.92, 1.58)	1.31 (0.97, 1.77)	1.95 (1.51, 2.52)***	0.57 (0.37, 0.88)*	1.04 (0.80, 1.37)
Middle	1.48 (1.15, 1.91)**	1.98 (1.57, 2.50)***	0.68 (0.46, 1.02)	1.00 (0.77, 1.30)	1.09 (0.81, 1.46)	1.50 (1.16, 1.93)**	0.61 (0.40, 0.92)*	0.86 (0.66, 1.12)
Richer	1.18 (0.92, 1.50)	1.33 (1.06, 1.68)*	0.93 (0.65, 1.33)	1.31 (1.01, 1.69)*	1.11 (0.84, 1.47)	1.41 (1.10, 1.81)**	0.93 (0.65, 1.33)	1.11 (0.85, 1.43)

Significant

*** p<0.001,

**p<0.01,

*p<0.05.

## Discussion

The study aims to explore the prevalence of stunting, wasting, underweight, and thinness among Bengali children under the age of five in Bangladesh and India, along with child-related, maternal, and socio-demographic factors on the effect of child undernutrition. The outcomes synthesized information on the nutritional status of Bengali children under the age of five in Bangladesh and India using BDHS and NFHS data, with the following significant findings: First, there was a relatively similar trend in nutritional status among under five Bengali children in Bangladesh and India, with stunting being the most serious problem in both countries, affecting roughly a third of the children, followed by underweight, wasting, and thinness. Second, one in three Bengali children was experiencing underweight in India, while in Bangladesh, the number was one in five, making childhood underweight a serious issue. Third, child undernutrition was linked to three key factors: maternal, child-related, and socio-demographic factors, where some factors coexisted in both countries, while others differed.

Wasting, underweight, and thinness differ significantly between these two countries, with the exception of stunting. However, for both Bangladesh and India, the distribution of z-scores suggests that the nutritional status of children were far below that of WHO-standards at each age and the gap increased as age increased. It is necessary to investigate whether the increase of gap is due to improper dieting after the first six month of breast-feeding. Even when compared to WHO-standard nutritional curve and studied population curve of stunting, wasting, underweight, thinness has identified a trend to undernutrition in each indicator. According to WHO, different forms of undernutrition have different effects, such as wasting conditions directly affecting the immunity system; stunting often leads to delayed mental development, poor school performance, and reduced intellectual ability; and being underweight increases the chance of death [26,27].

### Factors affecting child undernutrition in Bengalis

#### i. Child-related factors

In child and birth related factors, caesarean delivery reduced the risk of child undernutrition, and this condition was indirectly related to maternal health. Because several studies have indicated that cesarean delivery was more common in obese mothers when vaginal delivery not possible, and we found in this study that obese mothers’ children were less likely to suffer from undernutrition [28,29]. In terms of undernutrition, there were gender differences in children; girls fared better than boys. In Bangladesh, wasting and thinness were more prevalent in boys, whereas in India, only wasting was more prevalent. Child age was a significant determinant in stunting and underweight, particularly in children over the age of 23 months, showing that children did not receive adequate nourishment after quitting breastfeeding. Children born after the first child in Bangladesh have a higher risk of stunting.

#### ii. Maternal factors

Among the maternal factors, maternal undernutrition has been identified as a common factor of all kinds of child undernutrition for both countries. Similar finding was reported in other LMICs, in Uganda, Pakistan, Myanmar, and Nepal where these studies found that mothers who were normal or overweight have a lower risk of having undernourished children [30–33]. Prevalence of wasting and thinness were less among children of younger mothers in both countries. Maternal education has found as a common predictor of child undernutrition, with children of lower educated mothers having higher risk of undernutrition than the children of higher educated mothers. Other studies abroad in China, Indonesia, Nigeria, and Uganda have shown the coexistence of lower maternal education and child undernutrition [32,34–36]. Stunting and underweight were more common in Bangladeshi children whose mothers did not visit for ANC, while thinness was more common in India. Data from both countries show that ANC was associated to maternal education, with 20% of non-educated mothers not having a single ANC compared to 6.6% in educated mothers.

#### iii. Socio-demographic factors

Thinness was more prevalent among rural children in India. In both countries, about 53.3% of Bengali children were Muslims, with stunting being the most prevalent among them, while wasting, underweight, and thinness were less common than in others. Child undernutrition was also observed to be lower in small families. Stunting, wasting and underweight were more common in children from impoverished families than in children from wealthy families. Similar trend has been observed in Ethiopia, Indonesia, Uganda, and Papua New Guinea [32,34,37,38]. Li and colleagues investigated on undernutrition in children aged 12 to 59 months in 35 low- and middle-income countries (LMICs). The study indicated that the lack of maternal education, poorest household wealth and low maternal BMI coexist with child undernutrition [39].

In the present study, we have found that overall nutritional status of Bangladeshi children is better than Indian Bengali children. The nutritional status of Bengali under-five children appears to have improved in Bangladesh compared to India for a variety of reasons. Such as, infant and young child feeding (IYCF) practices indicate in Bangladesh, 35% of children (6–23 months) had a minimum acceptable diet (MAD) while in India it is 9.6% [40,41]. The MAD is an indicator that includes a composite of children fed with a minimum dietary diversity and meal frequency. In Bangladesh, the median duration of exclusive breastfeeding is 4.1 months and in India, it is 2.9 months among under-two children [40,41]. The DHS report found that young children in India consumed less micronutrient on average than children in Bangladesh. Among children aged 6–23 months, the proportion consuming food rich in vitamin A on the day or night before the interview has 76.6% in Bangladesh and 44.1% in India while consuming food rich in iron has 69.5% in Bangladesh and 17.9% in India, respectively [40,41]. In addition, the socio-economic conditions including the level of education of women in Bangladesh continue to improve rapidly. If all of these issues are specifically emphasised in the case of India, the nutritional disparity between the two country’s children will be reduced, and the future will be more prosperous for both countries.

### Key points for policy implications

The Bengali populations in Bangladesh and India are densely populated regions with over 1,000 people per square kilometer, making it challenging to ensure the health of Bengali children. To address this issue, both countries can adopt an inter-country plan to improve the health of Bengali children in the long term.

The food habits of the Bengali people in both countries are quite similar, which presents an opportunity to develop a balanced food list based on regional food production. However, the excessive use of chemicals in food production is causing a significant impact on the environment. As a result, soil and water pollution is increasing, which poses a threat to sustainable food production. Therefore, it is essential to focus on sustainable food production practices to eliminate the problem of malnutrition in the long run.

To ensure the sustainable production of food, it is crucial to give special attention to the environmental impact of food production practices. This can be achieved through the implementation of modern technology and the use of natural resources. Additionally, primary healthcare centers in both Bangladesh and India need to be modernized and expanded to improve access to healthcare for all individuals.

Moreover, there is a significant educational disparity between Bengali men and women, which has a direct impact on the health of Bengali children. In particular, women’s lack of access to education means they are less likely to have the knowledge and resources needed to make informed decisions about family planning. Additionally, the lack of education among women can lead to poor nutrition and health outcomes for their children, which can have long-lasting impacts on their physical and cognitive development. Therefore, special attention should be given to improving women’s education, which will play a crucial role in promoting family planning and child health. This could involve investing in girls’ education programs, providing scholarships and financial assistance to families who cannot afford to send their daughters to school, and implementing policies to encourage gender equality in the classroom. By enhancing women’s education, it will be possible to promote family planning, improve maternal and child health outcomes, and help break the cycle of poverty that often traps families in the region.

Enhancing the overall well-being of Bengalis is imperative, and this can be achieved by elevating their standard of living. By increasing the household wealth index of the community, many other problems can be effectively addressed. These may include issues related to poverty, unemployment, and social inequality.

Overall, a comprehensive approach is necessary to improve the health of Bengali children in Bangladesh and India. By prioritizing sustainable food production, modernizing primary health centers, enhancing mother’s education, and wealth index are key health challenges facing the region will be addressed, and all children will have the opportunity to grow up healthy and thrive.

### Strength and limitation of the study

The uniqueness of the study derives from the fact that this paper compares two communities; one predominantly follows Muslim faith and the other community predominantly follows Hindu faith that too from two different neighboring countries. The Bengali ethnic community has been given special attention in this study that extends beyond the nation’s territory. They primarily reside in India and Bangladesh, where undernutrition is a severe issue among under-five children. The findings of the study will assist policymakers in developing a separate strategy to prevent undernutrition, which will benefit the entire Bengali community. To the best of our knowledge, the two countries have never collaborated on a study with this similar objective. However, this study did not mention micronutrient deficiencies, and in the future research should focus on such concerns so that undernutrition in the Bengali children can be understood holistically.

## Conclusion

A comparative analysis was conducted in this study to evaluate the nutritional status of under-five Bengali between Bangladesh and India. The prevalence of stunting was more or less the same among children in Bangladesh and India. However, the prevalence of wasting, underweight and thinness were found to be higher in India than in Bangladesh. The problem of childhood underweight was especially severe among Indian Bengalis, with one out of every three Bengali children suffering from it. Stunting, which affects three out of every ten Bengali children, was the most prevalent form of undernutrition, followed by underweight, wasting, and thinness. This issue must be addressed with urgent and targeted interventions to ensure these children receive the proper nutrition and care they need to thrive and reach their full potential. Numerous factors play a role in determining the nutritional status of children, and present study has revealed that a mother’s level of education, mother’s BMI, and the household’s wealth index were showed prominent and significant influence. Wealth index and mothers’ BMI were supposed to be interlinked; both factors were found to have a substantial impact on a child’s nutritional well-being. Prioritizing and providing specific attention to mothers is crucial, as two of the three primary factors impacting child nutrition were closely associated with maternal care. The third factor, the wealth index, applies to all. It is mentionable that most of the problems might be solved if we want to improve standard of living (i.e. wealth index) of the people in the community. Thus, though the target groups are the children in both the countries, but the solution lies in the improvement of educational and nutritional status of mothers and would-be mothers. The onus rests with the planners to ensure how quickly we can achieve the four of the United Nations seventeen Sustainable Development Goals: no poverty, zero hunger, good health and well-being, and reduced inequality.

## Abbreviations

ANC: Antenatal care
AOR: Adjusted Odds Ratio
BAZ: BMI-for-age
BBS: Bangladesh Bureau of Statistics
BD: Bangladesh
BDHS: Bangladesh Demographic and Health Survey
BMI: Body Mass Index
CI: Confidence Interval
CIAF: Composite Index of Anthropometric Failure
DHS: Demographic and Health Surveys
HAZ: Height-for-age
IIPS: International Institute for Population Sciences
IN: India
IRB: Institutional Review Board
LMICs: Low-and Middle-Income Countries
NAF: No Anthropometric Failure
NFHS: National Family Health Survey
NIPORT: National Institute of Population Research and Training
No.: Number of frequency
ORG: Office of the Registrar General
SD: Standard Deviation
SDGs: Sustainable Development Goals
SPSS: Statistical Package for the Social Sciences
UN: United Nations
UNICEF: United Nations Children’s Fund
US: United States
VIF: Variance Inflation Factor
WAZ: Weight-for-age
WBG: World Bank Group
WHO: World Health Organization
WHZ: Weight-for-height
z-score: Nutritional z-score (derived from WHO-standard)
Z-value: Proportional Z-value (derived from statistical analysis)
